# The relationship between sex‐steroid hormones and brain volume variation in aging

**DOI:** 10.1002/alz.087762

**Published:** 2025-01-03

**Authors:** Thamires Naela Cardoso Magalhães, Trevor Bryan Jackson, Ivan Herrejon Gonzales, Jessica A Bernard

**Affiliations:** ^1^ Texas A&M University, College Station, TX USA; ^2^ Vanderbilt Memory & Alzheimer’s Center, Nashville, TN USA

## Abstract

**Background:**

Older females, particularly susceptible to Alzheimer’s disease (AD), may be affected by hormonal fluctuation during life. We aim to investigate the relationship between changes in brain volume and sex steroid hormones over time. We hypothesize that levels of sex hormones (17ß‐estradiol, progesterone, and testosterone) relate to changes in brain volume, especially in the hippocampus (HPC) and cerebellum (CB). The growing relevance of the CB in cognitive functions supports the exploration of potential associations in AD.

**Method:**

Subjects underwent structural magnetic resonance imaging (MRI) in conjunction with a series of cognitive and motor tasks. Roughly 12 months later, a subgroup of 91 participants—comprising 27 individuals in early middle age (average age 41±4.7 years; 13 females), 32 in late middle age (average age 58±4 years; 19 females), and 32 older adults (average age 72±6 years; 16 females)—underwent follow‐up MRI and task assessments (such as MoCA, digit, and symbol span). Saliva samples were collected during both visits for the quantification of sex hormones.

**Results:**

Analyzing longitudinal data with regression models that included all subjects, we found that baseline T and P levels were predictive of increased volumes in the bilateral cerebellar cortex and left HPC, respectively. In essence, individuals with higher levels of these basal hormones exhibited greater volumes over time. However, upon dividing the sample by sex, the result about P levels remained consistent in the female group. This suggests that, in females, higher baseline P levels continued to predict a greater volume in the left HPC (r = 0.351, p = 0.009; R^2^ = 0.57, p = 0.001). Upon closer examination of the groups independently, we identified positive correlations between baseline P levels and changes in volume (delta) of the left hippocampus (r = 0.441, p = 0.03) specifically in the middle‐aged group.

**Conclusion:**

Both T and P levels show a protective effect against age‐related structural changes. Ongoing analyses will explore this phenomenon further, with a focus on observing hormonal stages within the sample for clearer understanding.

